# Radiotherapy Increases Carotid Intima-Media Thickness in Patients with Nasopharyngeal Carcinoma Compared to a Healthy Control Group: A 6-Year Follow-Up Study

**DOI:** 10.3390/diagnostics15050528

**Published:** 2025-02-21

**Authors:** Yun-Fan Yvonne Chu, Ya-Yuan Hou, Wan-Chen Tsai, Chih-Cheng Huang, Hung-Chen Wang, Yu-Jih Su, Wei-Che Lin, Cheng-Hsien Lu, Nai-Wen Tsai

**Affiliations:** 1 Departments of Neurology, Chang Gung Memorial Hospital, Kaohsiung Branch, Kaohsiung City 833, Taiwan; yfchu98@gmail.com (Y.-F.Y.C.);; 2 Departments of Neurosurgery, Chang Gung Memorial Hospital, Kaohsiung Branch, Kaohsiung City 833, Taiwan; 3 College of Medicine, Chang Gung University, Taoyuan City 333, Taiwan; 4 Departments of Medicine, Chang Gung Memorial Hospital, Kaohsiung Branch, Kaohsiung City 833, Taiwan; 5 Departments of Radiology, Chang Gung Memorial Hospital, Kaohsiung Branch, Kaohsiung City 833, Taiwan; 6 Department of Biological Science, National Sun Yat-sen University, Kaohsiung City 804, Taiwan

**Keywords:** carotid plaque score, intima-media thickness, nasopharyngeal carcinoma, radiotherapy, mortality

## Abstract

**Background/Objective:** Vascular abnormalities are the primary histological changes in individuals undergoing radiotherapy for nasopharyngeal carcinoma (NPC). We sought to validate the hypothesis that the duration post-radiotherapy is linked to the progression of carotid intima-media thickness (IMT) and further explored its connection with mortality. **Methods:** Twenty-nine NPC patients who underwent radiotherapy and seventeen healthy controls were examined by carotid ultrasound for measurement of IMT and carotid plaque score at the common carotid artery (CCA), carotid bifurcation, and internal carotid artery, with follow-ups more than 6 years. **Results:** Initially, there was no discernible difference in internal carotid IMT between NPC patients and normal controls. However, a noteworthy increase in carotid IMT was observed after 6 years of radiotherapy (*p* < 0.0001). The carotid plaque score in NPC patients significantly exceeded that of the control group after 6 years (*p* < 0.0001). Linear regression demonstrated a positive correlation between carotid IMT and the duration post-radiotherapy. Logistic regression suggested that age and carotid IMT were predictors of mortality in NPC patients. **Conclusions:** Our study substantiates the positive correlation between carotid IMT and the duration of follow-up after radiotherapy. It found an increase in carotid IMT and plaque formation six years after radiotherapy compared to the control group. An increased carotid IMT may be correlated to an increased mortality rate and needs to be explored in future studies. Consequently, we recommend regular follow-up carotid ultrasonography for NPC patients undergoing radiation therapy.

## 1. Introduction

Nasopharyngeal carcinoma (NPC) represents a significant health concern and is recognized as one of the most prevalent cancers among the Chinese population. This malignancy is particularly common in individuals residing in the southeastern regions of China and Taiwan, where its incidence rates are reported to range between 20 and 40 cases per 100,000 person-years. This high prevalence has prompted numerous studies to better understand the disease’s etiology, risk factors, and effective treatment modalities [1,2]. The introduction and widespread adoption of combined chemotherapy and radiotherapy have markedly improved the prognosis of NPC patients by extending their overall survival rates. These treatment modalities have established themselves as the cornerstone of NPC management, especially for patients diagnosed in advanced stages of the disease [1].

As advancements in cancer treatment continue to evolve, there has been a paradigm shift in the way cancer is perceived within the broader scope of healthcare. Increasingly, cancer is being viewed not merely as an acute, life-threatening condition but rather as a chronic illness requiring long-term management and care. This perspective aligns with findings from our earlier research, which highlighted the pivotal role of oxidative stress and inflammation in contributing to chronic tissue damage induced by radiation exposure during cancer treatment [3,4]. Such damage, over time, underscores the necessity of monitoring and mitigating the long-term adverse effects associated with therapeutic interventions.

In clinical scenarios, vascular abnormalities emerge as some of the most prominent histological changes observed in patients following radiotherapy for head and neck cancers. These abnormalities often become evident within six months or more after the completion of radiation therapy and are recognized as a significant clinical concern due to their potential to compromise vascular health [5]. In particular, studies have focused on understanding the various factors linked to radiation-induced extracranial vasculopathy, a condition observed in NPC patients who have undergone radiotherapy. The identification and analysis of these factors provide crucial insights into the broader implications of radiation on vascular systems [6,7,8,9].

Among the markers used to assess vascular health, carotid intima-media thickness (IMT) has emerged as a reliable and widely accepted indicator of the severity of atherosclerotic disease [10]. Its measurement offers valuable information regarding the extent of vascular changes and serves as an early warning for potential cardiovascular complications [10,11]. Our earlier investigations further revealed the significant long-term effects of radiotherapy on carotid IMT in NPC patients. These findings emphasized the importance of monitoring carotid IMT as a part of the long-term follow-up care plan for individuals treated with radiotherapy [12,13].

Building on this foundation, the present study seeks to explore the intricate relationship between the duration of radiotherapy and its impact on the progression of carotid IMT in NPC patients. By examining this correlation in greater detail, this study aims to contribute to a deeper understanding of how radiation therapy affects vascular health over extended periods. The ultimate goal is to translate these findings into practical clinical applications, potentially enhancing both the immediate and long-term survival outcomes for patients undergoing radiotherapy. Such advancements in clinical practice could lead to better management strategies, ensuring that patients not only survive but thrive in their post-treatment lives.

## 2. Materials and Methods

### 2.1. Study Design

This research was structured as a case–control study and conducted at the Kaohsiung branch of Chang Gung Memorial Hospital. Recognized as one of Taiwan’s premier medical institutions and a referral center, this hospital is known for its excellence in patient care and research. Situated in the southern region of Taiwan, the hospital provides a comprehensive platform for advanced oncological research. This setting allowed for an in-depth analysis of nasopharyngeal carcinoma (NPC) and its associated complications.

### 2.2. Inclusion and Exclusion Criteria

Between January 2013 and August 2018, a total of 36 patients diagnosed with nasopharyngeal carcinoma (NPC), who were at least one year post-radiotherapy, were enrolled in the study. The study adhered to stringent ethical guidelines, receiving approval from the Ethics Committee of the hospital. All participants or their legally authorized representatives provided written informed consent.

To maintain the study’s integrity and ensure a focused investigation, several exclusion criteria were implemented. Patients were excluded if they (1) were less than one year post-radiotherapy, as this period is critical for acute recovery; (2) exhibited signs of fever or had a history of infection within the week preceding the study, which could confound inflammatory markers; (3) had undergone chemotherapy for cancer recurrence within the past three months, potentially altering vascular conditions; (4) had a medical history of significant comorbidities such as cerebral infarctions, coronary artery disease requiring angioplasty or bypass surgery, or renal failure necessitating dialysis before their NPC diagnosis and treatment; or (5) presented with conditions rendering IMT ultrasound measurements impractical, including severe fibrosis, extensive vascular calcification, or multiple plaque formations in the carotid arteries.

Ultimately, the study analyzed data from 29 participants with NPC. The control group consisted of 17 healthy volunteers matched for age and sex.

### 2.3. Diagnostic Criteria and Therapeutic Regimens of Nasopharyngeal Carcinoma (NPC)

The diagnosis of NPC was confirmed through histological evaluation by experienced pathologists specializing in head and neck oncology. Following the diagnostic process, a multidisciplinary approach was employed to optimize patient outcomes. Treatment protocols adhered to the NCCN Clinical Practice Guidelines in Oncology for Head and Neck Cancers established in the United States. Additionally, these protocols were adapted to the standards of the Kaohsiung CGMH Head and Neck Oncology Group at the Chang Gung Memorial Hospital Cancer Center.

Treatment regimens were stratified based on disease stage, classified using the American Joint Committee on Cancer (AJCC) staging system (2010 edition). For early-stage NPC (stages I–IIA), radiotherapy alone was deemed sufficient. In contrast, advanced stages (stages IIB–IV) necessitated a combined chemoradiation therapy (CCRT) approach to enhance treatment efficacy.

Radiotherapy was delivered in accordance with established protocols. The administered doses ranged from 68.4 to 75.6 Gy, depending on the stage of cancer. These doses were fractionated into 1.8 Gy per session, delivered five days a week. For CCRT, weekly intravenous infusions of cisplatin (40 mg/m^2^) were administered for five consecutive weeks in order to achieve a synergistic effect with radiation.

### 2.4. Clinical Assessment

A thorough medical and neurological evaluation was conducted for all participants. This included a detailed extracranial color-coded duplex sonography (ECCS) assessment, performed by trained professionals to evaluate vascular health comprehensively. The methodology for measuring the intima-media thickness (IMT) of the carotid artery was consistent with internationally recognized standards and previously published studies. This guaranteed the reliability and reproducibility of the measurements. In patients with NPC, ECCS was performed before CCRT, 6 months after CCRT, and 6 years after CCRT. The control group underwent ECCS at enrollment and 6 years after enrollment. Additionally, patient records were reviewed to document key variables, including body mass index (BMI), age at disease onset, and the duration of follow-up post-radiotherapy.

### 2.5. Assessment of Atherosclerosis

Vascular imaging was performed using a state-of-the-art B-mode ultrasound device (HDI 5000, Philips Healthcare, Bothell, WA, USA) equipped with a high-frequency linear array transducer (4–10 MHz). The procedure was conducted by a highly skilled and experienced technician blinded to the participants’ clinical histories, eliminating bias in image acquisition. Carotid intima-media thickness was measured using ultrasonography on the common carotid artery, carotid bifurcation, and internal carotid artery of both the left and right sides on sections devoid of plaques, spanning a 1 cm distance. On a longitudinal, two-dimensional ultrasound image, the anterior (near) and posterior (far) walls of the carotid artery appear as two bright white lines separated by a hypoechoic space. The intima-media thickness of the far wall is determined by measuring the distance between the leading edge of the first bright line (lumen–intima interface) and the leading edge of the second bright line (media–adventitia interface). For the near wall, this thickness is estimated as the distance between the trailing edges of the first and second bright lines [10]. The scanning protocol involved a systematic evaluation of the carotid arteries on both sides, focusing on a 1 cm segment proximal to the carotid bulb.

Special attention was given to imaging the far wall of the artery in a longitudinal plane. Plaques were identified as areas with significant thickening compared to the surrounding arterial wall. Plaque score is the number of plaques counted at the carotid bifurcation. These images were then processed using the QLAB software (QLAB, version 10, Philips Healthcare, Bothell, WA, USA) for precise IMT analysis in a procedure that blinded the analyst to the identity or history of the patient. The mean IMT value for each participant was calculated by averaging the measurements from both carotid arteries.

### 2.6. Biochemical Analysis

Blood samples were collected under standardized conditions to minimize variability and circadian influence. These samples were analyzed in the hospital’s central laboratory for various biochemical parameters, including lipid profiles, fasting blood glucose, HbA1c, and high-sensitivity C-reactive protein (hs-CRP). This analysis provided insights into the systemic metabolic and inflammatory states of the participants, contributing to the study’s understanding of atherosclerosis development.

### 2.7. Statistical Analysis

The statistical evaluation encompassed a multi-phase approach, executed using SAS software (version 9.1, SAS Institute, Cary, NC, USA). Data were expressed as means ± standard deviations (SDs) or medians with interquartile ranges, depending on the distribution. The chi-square or Fisher’s exact tests were employed for categorical data comparisons, while Student’s t-test was used for continuous variables. For non-normally distributed data, logarithmic transformation was applied before analysis.

1. Demographic analysis: The first phase involved comparing the demographic and clinical characteristics of NPC patients with healthy controls to identify baseline differences.
2. Risk factor evaluation: In the second phase, risk factors for carotid plaques in NPC patients were analyzed. Receiver operating characteristic (ROC) curves were generated for variables such as age and duration post-radiotherapy. The area under the curve (AUC) for each variable was calculated and compared.
3. Correlation analysis: The third phase assessed correlations between mean IMT and factors like age, gender, radiotherapy duration, cholesterol levels, and hs-CRP concentrations. Control subjects were assigned a radiotherapy duration value of zero for comparison.
4. Regression analysis: A two-step multiple linear regression analysis was conducted to determine the influence of various independent factors on the mean IMT. In the first model, variables significantly associated with mean IMT were identified. These variables were then further examined in a second model to confirm their independent effects on IMT.

## 3. Results

### 3.1. Baseline Characteristics of the Study Patients

The baseline demographic and laboratory characteristics of the participants, including both NPC patients (*n* = 29) and healthy volunteers (*n* = 17), are summarized in Table 1. The study groups were carefully matched to assure comparability, particularly with respect to critical variables such as age, gender, and body mass index (BMI). Statistical analysis confirmed that there were no significant differences between the two groups for these baseline parameters.

In terms of hematological parameters, including white blood cell (WBC) counts, red blood cell (RBC) counts, and platelet counts, no statistically significant differences were observed between the NPC group and the control group. This finding underscores that the groups were comparable regarding basic blood cell profiles.

However, significant disparities were identified in several biochemical markers. Notably, NPC patients exhibited markedly elevated levels of glycosylated hemoglobin (HbA1c), triglycerides, and high-sensitivity C-reactive protein (hs-CRP) compared to healthy controls. These elevations were statistically significant, with a *p*-value of less than 0.05. These findings suggest that NPC patients may be at a heightened risk of metabolic and inflammatory dysregulation, likely attributable to the long-term effects of radiotherapy and the underlying disease process.

### 3.2. Long-Term Follow-Up of CCA IMT and Plaque Score Between NPC Patients with Radiotherapy and Controls

An analysis of the long-term vascular outcomes revealed critical insights into the effects of radiotherapy on carotid intima-media thickness (IMT) and plaque scores in NPC patients. At baseline, carotid IMT and plaque scores showed no conspicuous differences between the NPC patients and healthy controls, as shown in Table 2.

**Figure 1:**
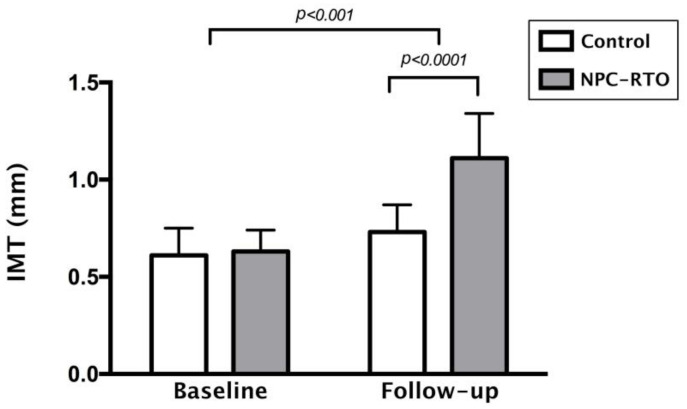
Comparison of IMT between NPC patients with RTO and controls. (IMT: intima-media thickness; NPC: nasopharyngeal carcinoma; RTO: radiation therapy oncology).

However, over an extended follow-up period, significant changes became apparent. After a 6-year duration post-radiotherapy, carotid IMT measurements in NPC patients demonstrated a substantial increase compared to the control group. This increase was highly statistically significant, with a *p*-value of less than 0.0001, as illustrated in Figure 1. The rise in carotid IMT over time highlights the progressive nature of vascular changes induced by radiation exposure.

**Figure 2:**
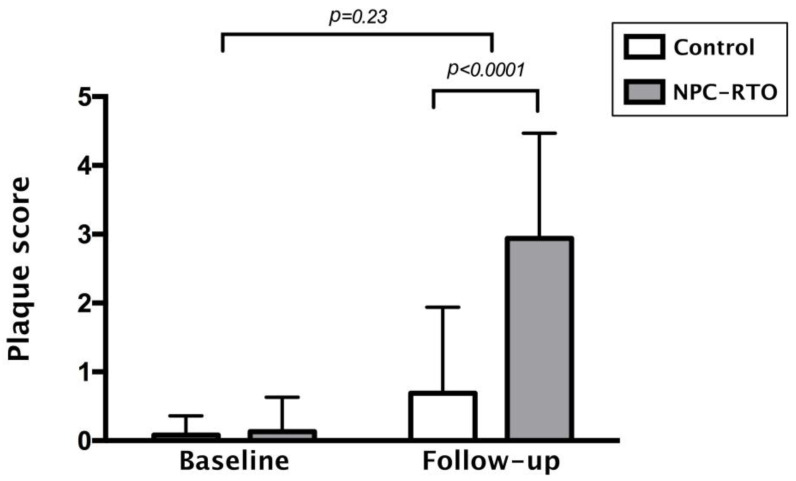
Comparison of plaque score between NPC patients with RTO and controls. (NPC: nasopharyngeal carcinoma; RTO: radiation therapy oncology).

In parallel, the carotid plaque scores also exhibited a notable and statistically significant increase among NPC patients compared to controls after the 6-year follow-up period (*p* < 0.0001), as depicted in Figure 2. These findings underscore the cumulative impact of radiotherapy on the structural integrity of the carotid arteries, with long-term implications for atherosclerosis development and cardiovascular risk.

**Figure 3:**
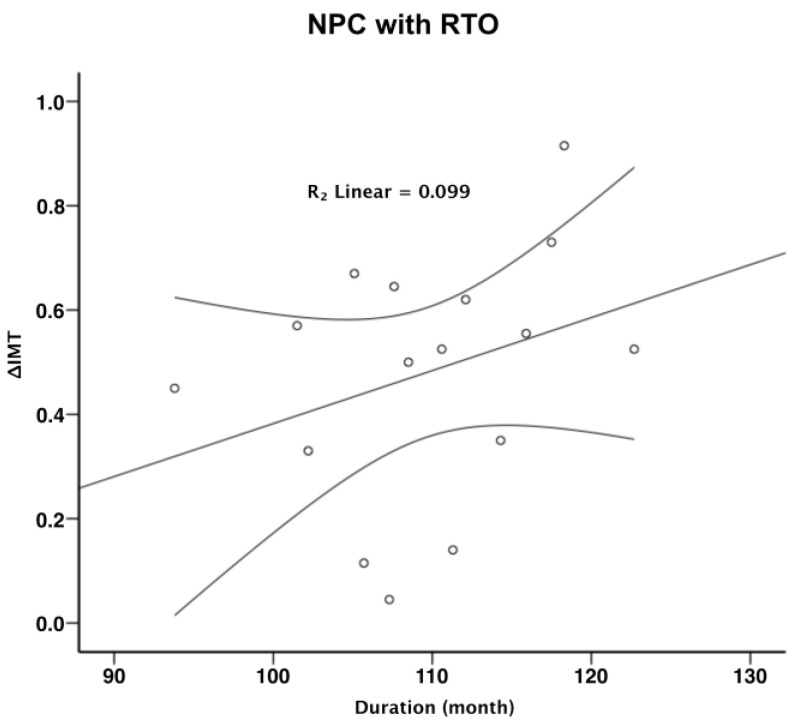
Partial correlation between the change in IMT (ΔIMT) and duration in NPC patients. (IMT: intima-media thickness; NPC: nasopharyngeal carcinoma).

**Figure 4:**
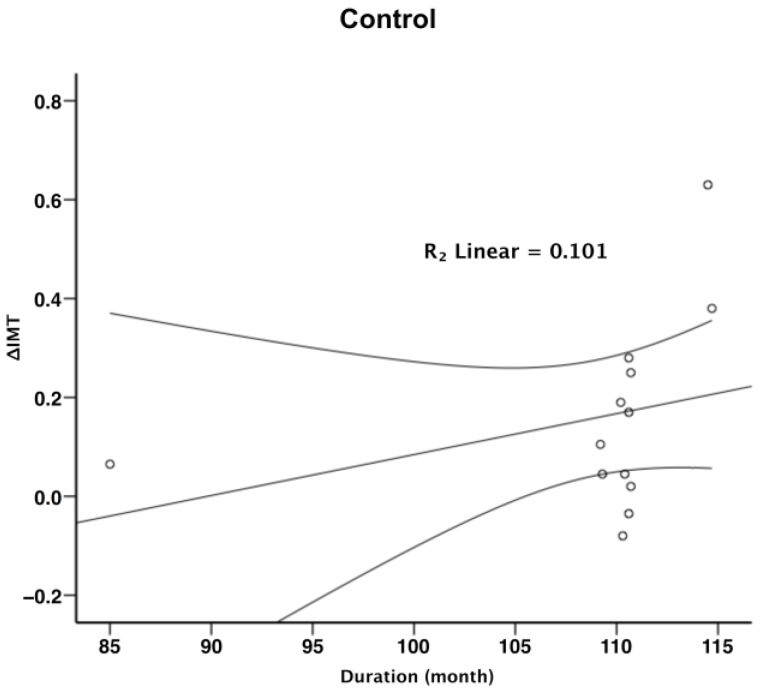
Partial correlation between the change in IMT (ΔIMT) and duration in controls. (IMT: intima-media thickness).

To further explore the relationship between radiotherapy duration and carotid IMT, a linear regression analysis was conducted. The results revealed a positive correlation between the duration of post-radiotherapy and carotid IMT, as demonstrated by R^2^ linear values of 0.099 and 0.101 (Figure 3 and Figure 4, respectively). This relationship underscores the significance of monitoring vascular health in NPC patients over time, as the risk of progressive arterial thickening increases with the duration following radiation therapy.

### 3.3. Prognostics Factors of Mortality in NPC Patients After Radiotherapy

The study also sought to identify key prognostic factors associated with mortality in NPC patients who had undergone radiotherapy. These findings are presented in Table 3. Through logistic regression analysis, age and carotid IMT emerged as significant predictors of mortality in this patient population. Specifically, both variables demonstrated statistically significant associations with mortality risk, with a *p*-value of 0.01 and 0.007, respectively.

## 4. Discussion

In this study, we conducted an in-depth investigation of the intima-media thickness (IMT) and plaque score of the common carotid artery (CCA) in patients with nasopharyngeal carcinoma (NPC) who had undergone radiotherapy. The cohort was followed for an extended period exceeding 6 years to explore the long-term vascular effects of radiation therapy. Our research yielded two findings. Firstly, a strong and statistically significant positive correlation was observed between carotid IMT and the duration of follow-up post-radiotherapy. This finding suggests that radiation-induced vascular changes are progressive and accumulate over time. Secondly, advanced age and increased carotid intima thickness were observed to be potential prognostic factors associated with mortality in NPC patients following radiotherapy. These results underscore the importance of long-term monitoring and the need to address vascular health in cancer survivors undergoing radiotherapy.

Radiation-induced vasculopathy emerged as a key finding in this study, demonstrating that this condition is not static but rather evolves as time progresses after radiotherapy. The correlation between longer follow-up durations and increased carotid IMT highlights the ongoing nature of vascular damage initiated by radiation exposure. This phenomenon aligns with prior studies that have described radiation therapy as a double-edged sword—it effectively eradicates cancer cells but also induces damage to the surrounding healthy tissues, particularly the vasculature. The carotid arteries, being in close anatomical proximity to radiation-targeted regions in the neck, are especially susceptible to this collateral damage.

In clinical settings, vascular abnormalities are among the most common histological alterations observed in patients receiving radiation therapy for head and neck cancers. These changes typically begin to manifest more than 6 months post-irradiation [14,15,16,17].

Radiation-induced damage to the carotid arteries has far-reaching implications for the long-term health of NPC survivors. Atherosclerotic and thrombotic complications following radiation therapy are well documented in the literature and represent a critical area of concern [18,19,20,21,22,23]. In a prior study involving 910 patients who survived at least 5 years after undergoing radiation therapy for head and neck tumors, a 6% incidence of stroke and a clinically significant carotid stenosis rate of 17% were reported [24]. These findings underscore the importance of addressing vascular complications in this patient population. Carotid IMT, a marker of atherosclerosis and vascular health, may be associated with mortality. Our results emphasize the role of radiation-induced vascular damage as a contributor to long-term outcomes in NPC patients.

Radiation-induced atherosclerosis arises from a complex interplay of cellular and molecular mechanisms. It involves the accumulation of lipids, inflammatory cells, and extracellular matrix components, with vascular smooth muscle cells contributing to plaque development. Radiation therapy exacerbates this by causing endothelial damage, inflammation, and oxidative stress, advancing plaques to calcification or rupture. Acute complications, such as myocardial infarction and stroke, may result from plaque rupture or thromboembolism [25,26,27].

In our study, the progressive increase in carotid IMT over a 6-year period suggests a cumulative impact of radiation on vascular health. This finding aligns with the hypothesis that radiation accelerates the natural aging process of the vasculature, leading to premature atherosclerosis [23,27,28]. The linear regression analysis performed in our study further supports this notion, as a direct relationship was observed between the duration post-radiotherapy and carotid IMT. This relationship highlights the importance of duration as a critical factor in assessing radiation-induced vascular changes.

Furthermore, our study identified age and carotid IMT as likely predictors of mortality in NPC patients who underwent radiotherapy. Age has long been recognized as a non-modifiable risk factor for cardiovascular diseases, and its role in this context likely reflects the cumulative burden of aging on vascular and overall health [29]. On the other hand, carotid IMT serves as a surrogate marker for subclinical atherosclerosis and is predictive of future cardiovascular events [30,31,32]. The inclusion of carotid IMT as a potential mortality predictor emphasizes the need for early detection and management of vascular changes in this patient population.

These findings have important clinical implications. First, they highlight the need for routine vascular health monitoring in NPC survivors who have undergone radiotherapy. Second, these results underscore the importance of a multidisciplinary approach in the long-term care of NPC survivors. Collaboration between oncologists, neurologists, cardiologists, and primary care physicians is essential to address the complex interplay between cancer treatment and cardiovascular health. Future research should focus on developing strategies to prevent or mitigate radiation-induced vascular damage, thereby enhancing the quality of life and survival rates of cancer survivors.

### Limitations

This study, while providing some insights into the long-term effects of radiotherapy on carotid intima-media thickness (IMT) and atherosclerotic changes in nasopharyngeal carcinoma (NPC) patients, has several limitations that should be acknowledged to ensure a balanced interpretation of the findings. These limitations highlight areas where caution is warranted when generalizing the results and suggest directions for future research.

Firstly, we did not exclude patients with underlying comorbidities such as diabetes mellitus and hyperlipidemia, both of which are well-established risk factors for atherosclerosis and cardiovascular diseases. The presence of these conditions in some participants might have introduced additional variability into the data, potentially confounding the relationship between radiotherapy and vascular changes. For instance, diabetes mellitus is known to accelerate atherosclerotic processes through mechanisms involving chronic inflammation, oxidative stress, and endothelial dysfunction. Similarly, hyperlipidemia contributes to plaque formation through lipid accumulation and foam cell development. These factors may have compounded the observed effects of radiotherapy, making it challenging to isolate the specific impact of radiation on carotid IMT.

Secondly, many participants were taking medications that could influence vascular health and atherosclerosis progression. These medications included statins, which are widely prescribed for managing hyperlipidemia and are known to have pleiotropic effects, such as reducing vascular inflammation and stabilizing plaques. Calcium channel blockers, another commonly used class of drugs, can improve vascular compliance and reduce arterial stiffness, potentially mitigating some of the adverse vascular effects of radiotherapy. The inclusion of patients on these medications might have introduced variability in the observed outcomes, as their protective effects could partially counteract the radiation-induced vascular damage. Without stratifying or adjusting for the use of these medications, it is difficult to determine the true magnitude of radiotherapy’s impact on carotid IMT and plaque progression.

Additionally, this study did not account for lifestyle factors that could influence vascular health, such as diet, physical activity, and smoking status. Smoking, for example, is a major risk factor for both atherosclerosis and cancer, and its synergistic effects with radiation therapy could have exacerbated vascular damage. On the other hand, a healthy diet and regular physical activity may have provided protective effects against radiation-induced vascular changes. The lack of detailed data on these variables limits our ability to fully contextualize the findings within the broader scope of cardiovascular risk factors.

Another important limitation is the inherent challenge of evaluating the effect of radiotherapy on IMT progression within the same individual. This limitation restricts our ability to draw definitive causal conclusions about the temporal relationship between radiotherapy and vascular changes. Instead, we relied on cross-sectional comparisons and longitudinal follow-up data, which, while informative, may not capture the full spectrum of radiation-induced vascular dynamics.

Furthermore, the study cohort was drawn from a single institution with a limited number of participants, which may limit the generalizability of the findings to broader populations. Differences in radiation techniques, dosimetry, and patient demographics at other centers could influence the applicability of the results. For example, advances in radiotherapy techniques, such as intensity-modulated radiotherapy (IMRT) and proton therapy, aim to minimize collateral damage to surrounding tissues, including the carotid arteries. Patients treated with these modern modalities may experience different vascular outcomes compared to those in our study.

Lastly, our study did not include a detailed analysis of genetic or molecular markers that could provide insights into individual susceptibility to radiation-induced vascular damage. Genetic predispositions, such as variations in genes related to oxidative stress, inflammation, or DNA repair, may influence the extent of vascular injury following radiotherapy. Understanding these factors could help identify high-risk patients who might benefit from tailored preventive or therapeutic interventions.

In summary, while this study sheds light on the long-term vascular effects of radiotherapy in NPC patients, the limitations mentioned above underscore the need for further research. Future studies should aim to account for and adjust for underlying comorbidities, medication use, lifestyle factors, and genetic predispositions. Prospective longitudinal designs with pre-treatment baselines and multicenter collaborations would provide more robust data, enabling a clearer understanding of the relationship between radiotherapy and vascular health.

## 5. Conclusions

This study underscores the progressive nature of radiation-induced vascular changes in nasopharyngeal carcinoma (NPC) patients, evidenced by a positive correlation between carotid intima-media thickness (IMT) and the duration of follow-up post-radiotherapy. Importantly, age and increased carotid IMT potentially served as predictors of mortality in this population, highlighting the long-term cardiovascular risks associated with radiation therapy. These findings emphasize the critical need for regular monitoring of carotid IMT through ultrasonography as a part of the routine follow-up care for NPC patients; however, it requires further full-scale studies to provide concrete evidence.

Given the progressive nature of these vascular changes, early detection and intervention could play a pivotal role in mitigating long-term morbidity and mortality. Carotid ultrasonography not only serves as a non-invasive tool for assessing subclinical atherosclerosis but also provides valuable prognostic insights that can inform personalized management strategies. Integrating vascular health assessments into the follow-up care of NPC patients may help address the dual challenges of cancer survivorship and cardiovascular disease prevention.

While this study provides crucial insights, larger, multicenter trials are urgently needed to validate these findings and expand our understanding of radiation-induced vascular damage in NPC patients. Such trials could explore the underlying mechanisms, evaluate the impact of modern radiotherapy techniques, and identify optimal screening intervals and therapeutic strategies to minimize cardiovascular complications. These efforts would contribute to the development of evidence-based guidelines for improving long-term outcomes and quality of life for NPC survivors.

## Figures and Tables

**Table 1:** Baseline characteristics between patients with NPC and controls.

	Controls (*n* = 17)	NPC (*n* = 29)	*p*-Value
Age			
(year; mean ± SD)	48.4 ± 12.0	55.1 ± 12.3	0.197
Sex			
(*n*; male/female)	8/9	14/15	0.97
BMI			
(mean ± SD)	22.6 ± 2.8	24.8 ± 3.3	0.009 *
Laboratory data			
White blood cells (×10^3^/mL)	5.7 ± 1.9	6.3 ± 2.0	0.191
Red blood cells (×10^6^/mL)	23.0 ± 2.0	25.0 ± 1.8	0.88
Platelet counts (×10^4^/mL)	222.6 ± 51.2	223.0 ± 67.1	0.98
hs-CRP (mg/L)	1.2 ± 0.9	7.8 ± 10.3	0.0018 *
Total cholesterol (mg/dL)	191.9 ± 28.0	189.3 ± 36.6	0.763
LDL cholesterol (mg/dL)	106.7 ± 25.1	108.9 ± 29.8	0.760
Triglycerol (mg/dL)	83.9 ± 35.4	137.8 ± 70.0	0.001 *
HbA1c (%)	5.5 ± 0.2	6.2 ± 1.2	0.003 *

* Statistically significant compared to controls (*p* < 0.05). HbA1c, glycated hemoglobin; hs-CRP, high-sensitivity C-reactive protein; LDL, low-density lipoprotein; SD, standard deviation; BMI, body mass index.

**Table 2:** Change in intima-medium thickness and plaque score between patients of NPC with radiotherapy and controls.

	Controls (*n* = 17)	NPC (*n* = 29)	*p*-Value
Baseline			
RIMT	0.60 ± 0.09	0.64 ± 0.67	0.232
LIMT	0.64 ± 0.16	0.71 ± 0.21	0.185
IMT-mean	0.62 ± 0.12	0.68 ± 0.16	0.148
Plaque score	0.15 ± 0.46	0.19 ± 0.53	0.716
6-year follow-up			
RIMT	0.70 ± 0.13	1.05 ± 0.26	<0.0001 *
LIMT	0.74 ± 0.17	1.18 ± 0.27	<0.0001 *
IMT-mean	0.72 ± 0.14	1.11 ± 0.23	<0.0001 *
Plaque score	0.69 ± 1.25	2.94 ± 1.52	<0.0001 *

* Statistically significant compared to controls; *p* < 0.05. IMT, intima-media thickness; RIMT, right intima-media thickness; LIMT, left intima-media thickness.

**Table 3:** Prognostic factors of mortality in patients with NPC after radiotherapy.

	Mortality (*n* = 10)	Survival (*n* = 19)	*p*-Value
Age (year)	63.3 ± 8.5	51.2 ± 12.2	0.01 *
Sex (*n*; male/female)	4/6	9/10	0.85
BMI	24.7 ± 2.9	24.8 ± 3.6	0.91
Laboratory data			
White blood cells (×10^3^/mL)	6.5 ± 2.2	6.3 ± 2.0	0.73
Red blood cells (×10^6^/mL)	4.4 ± 0.6	4.6 ± 0.8	
Platelet counts (×10^4^/mL)	206.6 ± 79.5	229.3 ± 62.3	0.37
hs-CRP (mg/L)	4.1 ± 1.1	3.0 ± 3.7	0.68
Total cholesterol (mg/dL)	193.5 ± 33.0	187.7 ± 38.4	0.68
LDL cholesterol (mg/dL)	116.0 ± 22.3	106.2 ± 32.2	0.38
Triglycerol (mg/dL)	140.0 ± 54.8	137.0 ± 75.9	0.91
HbA1c (%)	6.7 ± 1.5	6.1 ± 1.0	0.20
IMT and plaque score			
Before RTO			
RIMT	0.75 ± 0.24	0.60 ± 0.11	0.093
LIMT	0.82 ± 0.22	0.67 ± 0.19	0.042
IMT-mean	0.79 ± 0.19	0.64 ± 0.13	0.009 *
Plaque score	0.40 ± 0.70	0.12 ± 0.43	0.253
6-months after RTO			
RIMT	0.93 ± 0.28	0.66 ± 0.19	0.015 *
LIMT	0.96 ± 0.17	0.69 ± 0.19	0.008 *
IMT-mean	0.96 ± 0.17	0.69 ± 0.19	0.007 *
Plaque score	0.40 ± 0.70	0.12 ± 0.43	0.253

The data were presented with mean ± SD. * Statistically significant compared between groups (*p* < 0.05). BMI, body mass index; HbA1c, glycated hemoglobin; hs-CRP, high-sensitivity C-reactive protein; LDL, low-density lipoprotein; IMT, intima-media thickness; RIMT, right intima-media thickness; LIMT, left intima-media thickness.

## Data Availability

Data are available under reasonable request to the corresponding author.
